# Editorial

**Published:** 1853-01

**Authors:** 


### THE MEDICAL EXAMINER.

PHILADELPHIA, JANUARY, 1853.

The press of original matter in the present number, (much of which has been delayed from previous numbers,) has so circumscribed our space, that we must forego our usual salutation to our readers, on the commencement of a new volume. We must, however, express our acknowledgements for the steadily increasing support with which our editorial labors have been encouraged; and we may also briefly add, that we shall relax no efforts to maintain the position of the Examiner in every department—original, bibliographical, clinical, and selected. If the usual limits of the last have been somewhat curtailed of late, we trust that our readers have been repaid for the temporary omission, in the value of our original matter and the elaborate character of our bibliographical notices; and we beg to assure them that the abridgement of the 'Record' is only temporary.

